# Recent Advances in Natural Polyphenol Research

**DOI:** 10.3390/molecules27248777

**Published:** 2022-12-11

**Authors:** Irene Dini, Lucia Grumetto

**Affiliations:** Department of Pharmacy, University of Naples Federico II, Via Domenico Montesano 49, 80131 Napoli, Italy

**Keywords:** antioxidant, circular economy, agri-food wastes, sustainability, flavonoids, polyphenols bioavailability, polyphenols activity, functional food, nutraceuticals, cosmeceuticals, nano-delivery, bioavailability, health

## Abstract

Polyphenols are secondary metabolites produced by plants, which contribute to the plant’s defense against abiotic stress conditions (e.g., UV radiation and precipitation), the aggression of herbivores, and plant pathogens. Epidemiological studies suggest that long-term consumption of plant polyphenols protects against cardiovascular disease, cancer, osteoporosis, diabetes, and neurodegenerative diseases. Their structural diversity has fascinated and confronted analytical chemists on how to carry out unambiguous identification, exhaustive recovery from plants and organic waste, and define their nutritional and biological potential. The food, cosmetic, and pharmaceutical industries employ polyphenols from fruits and vegetables to produce additives, additional foods, and supplements. In some cases, nanocarriers have been used to protect polyphenols during food processing, to solve the issues related to low water solubility, to transport them to the site of action, and improve their bioavailability. This review summarizes the structure-bioactivity relationships, processing parameters that impact polyphenol stability and bioavailability, the research progress in nanocarrier delivery, and the most innovative methodologies for the exhaustive recovery of polyphenols from plant and agri-waste materials.

## 1. Introduction

Natural polyphenols are secondary metabolites of plants, vegetables, cereals, fruits, coffee, tea, and other plants. The exceptional functionality and biocompatibility of the polyphenols have stimulated the interest of researchers to use them as building blocks in functional foods, supplements, cosmetics, and drugs [1,2]. They have a phenolic ring, a basic monomer responsible for the protective action against oxidative injury [3]. Polyphenolic compounds can moderate oxidative stress and prevent or even inhibit oxidation by chelating iron and scavenging reactive radicals [4,5]. Dietary polyphenols can act as antioxidants, anti-inflammatory, and antiallergic compounds, decrease and prevent age-related diseases, can help against cardiovascular events (i.e., through their hypocholesterolemic, anti-thrombotic, antihypertensive, and anti-atherogenic), cancer, osteoporosis, diabetes, and neurodegenerative diseases [6]. The dietary polyphenols’ bioavailability depends on the chemical and physical characteristics of the natural matrix that contains them, the stability during the digestive process, the intestinal enzymes’ metabolization, and intestinal microbiota [7]. The gut microflora can modify the polyphenols’ bioactivity and bioavailability [8]. Their bioaccessibility can be affected by preservation and processing methods, the interaction with the matrix components, and the fluids and enzymes secreted during digestion [9]. Physical, chemical, and enzymatic treatments can alter their properties. The preservation and processing can determine damage to the native polyphenol molecules and the production of new “process-derived” compounds [10]. Nanodelivery technology can improve polyphenols’ absorption, bioavailability, functional quality, and performance [11,12,13,14,15].

This review summarizes the natural polyphenols classes, the extraction and methods performed to isolate them from natural sources and agro-waste, the factors that affect their bioavailability, and the application and development of nanodelivery systems.

## 2. Polyphenols in Nature

**Figure 1:**
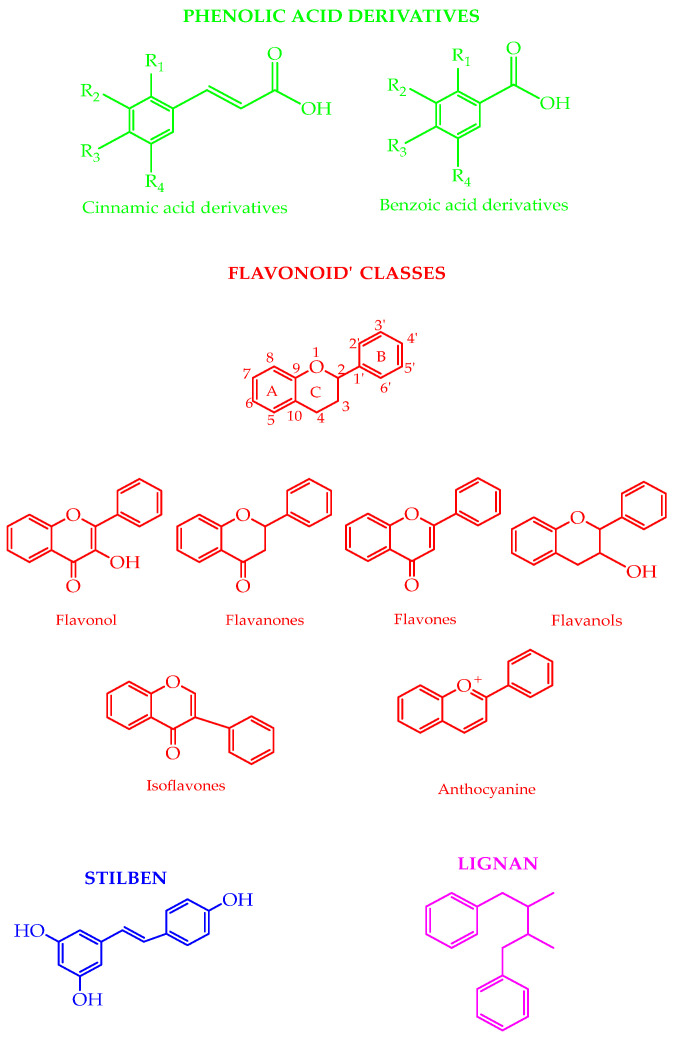
Chemical structures of the different polyphenol classes. The colors indicate the sub-class. The numbers indicate the positions in the nomenclature.

Polyphenols are involved in plant defense against pathogens and ultraviolet radiation [16]. The plants’ outer layers contain higher phenolics [17]. Insoluble phenolics are in cell walls, while soluble phenolics are in the plant cell vacuoles [18]. The degree of ripeness during harvest time, pedoclimatic conditions, infections, processing, and storage can affect the polyphenolic content [19]. The phenolic acids (e.g., derivatives of cinnamic acid and benzoic acid), flavonoids (e.g., flavonols, flavanones, flavones, flavanols, isoflavones, and anthocyanins), lignans, and stilbenes are the most naturally occurring classes of compounds (Figure 1). The shikimate pathway produces the phenolic acids. The phenylpropanoids pathway forms lignans, lignins, flavonoids, and stilbenes [20,21]. The biosynthesis of complex polyphenols is linked to primary metabolism: the flavonoids’ ring B and the chromane ring originate from the amino acid phenylalanine, obtained from the shikimate pathway, whereas ring A is formed from three malonyl-CoA units added through sequential decarboxylation condensation reactions [22]. In food, polyphenolic compounds can impact astringency, bitterness, flavor, color, and oxidative stability [19].

## 3. Polyphenols Bioavailability

There is no relation between the concentration of polyphenols in food and their bioavailability in the human body. The polyphenols, after ingestion, pass through the gastrointestinal epithelium and enter the circulatory vessels to reach the site of action. In food, polyphenols can exist as aglycon, glycosides, esters, or polymers.

The polyphenols’ chemical structure limits the rate, absorption, and metabolites circulating in the plasma. The polyphenolic compounds with hydroxyl groups can be modified by methylation, glucuronidation, or sulfation enzymatic reactions. The 5–10% of total polyphenolic compounds may be metabolized in the small intestine. The rest of the polyphenols accumulate in the large intestine and are evacuated in the feces [23]. The conjugated polyphenols must be hydrolyzed by colonic microflora or intestinal enzymes (i.e., β-glucosidases and lactase-phlorizin hydrolase) before absorption [24]. During the absorption process, they are transformed into oligomeric phenols by gastric acid in the stomach, and glycosidic polyphenols are cleaved by cytosolic glucosidase and lactase in the small intestine into aglycon and glycoside(s) (e.g., glucose, xylose, and galactose) radicals [25]. Finally, intestinal bacterial enzymes can metabolize the remaining aglycone fraction. In the intestinal and colonic epithelium, polyphenols can be involved in conjugation reactions with methyl, glucuronide, or sulfate groups, making the identification of the metabolites in the blood and tissue complex [26]. The glycosides of quercetin and the isoflavones (genistein and daidzein) are not recovered in plasma or urine [27,28,29]. Instead, anthocyanins glycosides are the most representative circulating forms [30,31]. Experimental studies showed that quercetin, without glycosides, is absorbed at the gastric level [32], anthocyanins in the stomach [33,34], and proanthocyanidins [33,35] and hydroxycinnamic acids are absorbed by the small intestine [36]. The remaining polyphenols are hydrolyzed in the colon by microflora enzymes into aglycones that are metabolized into benzoic acid derivatives [37,38].

The polyphenols’ digestibility affects their biological properties [39]. Soluble polyphenols have more evident responses during gastrointestinal digestion since the cell wall does not protect them. Unfortunately, human enzymes cannot digest some cell wall materials.

The flavonoids linked to other macromolecules cannot exert their beneficial actions [37]. The heat and pressure application (processing parameters) can facilitate the disruption of the cell wall and their release improving their bioactivity [38].

The pH and number of OH groups in benzene rings can affect phenolic stability. Conjugated nonphenolic aromatic acids, such as trans-cinnamic acid, are stable at high pH. The aromatic acids with a single OH group (e.g., ferulic acid) are stable at high pH because they do not form quinone oxidation products. The aromatic acids, with two phenolic OH groups (e.g., caffeic acid) or three (e.g., gallic acid), are unstable at pH 7–11. The changes are ascribable to the two adjacent phenolic OH groups attached to the benzene ring. Flavonoid molecules (e.g., rutin) that have a wholly conjugated aromatic structure are influenced by pH because the spatial arrangement between the π-electron system and an OH group controls the extent of π-orbital overlap and susceptibilities to the chemical change. The flavonoids in which the first benzene ring is located in the meta-position (e.g., catechin, epicatechin) do not have planar structures. Therefore, the π-electrons of the two benzene rings cannot cooperate via conjugation and are less susceptible at high pH [40,41].

In plant-based food, polyphenols and cell wall polysaccharides co-exist, and their affinity may influence foods’ physicochemical and nutritional properties during processing and digestion. The affinity of cell wall polysaccharides with polyphenols depends on their structures, concentrations, temperature, pH, ionic strength, and the presence of proteins [42].

The enzyme concentrations, solubility, pH, digestion time [43], and processing methods (e.g., washing, refrigerating, fermentation, grain milling, roasting, juicing, blanching, and thermal processing) impact polyphenols’ bioaccessibility and absorption [9].

Cooking and freezing processes positively impact the polyphenols’ bioaccessibility since they soften the cell wall. The cooking medium also influences their bioaccessibility [44,45].

Pasteurization affects the polyphenols’ bioaccessibility in the function of the heat treatment intensity, steps involved in processing, and type of food, decreasing the adverse processing effects on small bioactive compounds and even increasing polyphenols content [46]. Pasteurization can enhance food polyphenols extraction since the temperature softens the cell wall [47].

Finally, the interactions between macronutrients, micronutrients, and other phytochemicals, in finished products may also impact polyphenols’ stability [48].

## 4. Polyphenols & Microbiota

Gut microbiota can break the flavonoid C-ring in different positions, producing simple phenolics from the A and B rings. Most of these metabolites are acid or aldehyde phenolics with 1, 2, and (or) 3 hydroxyl and methyl ester radicals. Non-flavonoid phenolics (e.g., hydrolyzable tannins, stilbenes, lignans, and hydroxy-benzoic acid derivatives) are absorbed in the small intestine based on their chemical complexity. The gut bacteria can hydrolyze the ester bonds in tannins, dehydroxylate, and decarboxylate, the gallic acid [49], and reduce the resveratrol and its precursors [50].

The role of polyphenols and their metabolites on the gut microbiota is not elucidated. They probably have a prebiotic-like effect [51], since they can modulate the gut microbial profile. [52,53].

The polyphenols’ prebiotic effect is associated mainly with the promotion of probiotics (e.g., Bifidobacteriaceae and Lactobacillaceae) or the inhibition of pathogenic bacteria (i.e., *E. coli*, *Clostridium perfringens*, and *Helicobacter pylori* [52]) resulting in reduced proinflammatory immune response and decreased risk of colon cancer, gastroenteritis, inflammatory bowel disease, and metabolic syndrome [54,55].

Some polyphenols prevent bacterial growth, binding the cell membranes in a concentration-dependent manner. Catechins can change the microbial (i.e., *Bordetella bronchiseptica*, *Klebsiella pneumonie*, *E. coli*, *Pseudomonas aeruginosa*, *Serratia marcescens*, *Bacillus subtilis Salmonella choleraesis*, and *Staphylococcus aureus*) cell membrane permeability by producing H_2_O_2_ [56]. In Gram-positive bacteria, polyphenolic compounds can delay the oligopeptides autoinducers that sense the bacterial quorum sensing. In Gram-negative bacteria, they can prevent the bacteria-acylated homoserine lactones autoinducers [57].

## 5. Effects of the Food-Processing Techniques on Polyphenol Levels and Bioavailability

The heat treatments (e.g., boiling, steaming, frying, stewing, baking, roasting, ovens, steam, and microwave) and the transformation food processing (e.g., roasting, toasting, drying, pasteurization, canning, and sterilization), can affect the polyphenols’ bioavailability. The heat breaks cell walls, mobilizes the phenolic compounds, improves their availability, enhances their oxidation processes, and degrades them based on their thermostability. Domestic cooking and industrial thermal processes can cause losses in polyphenols, with significant variability depending on the nature of food matrices [58].

Boiling produces the most harmful polyphenol composition changes. Instead, steaming and frying can preserve them since the polar media (water) can extract higher levels of polyphenol than nonpolar media (oil) [59]. During boiling, heat decomposes the tissues, and the phenolics leak into the water [60]. Water volume can impact the polyphenol alteration during the heat process: small water volumes produce lower phenolic extraction than larger volumes [61]. Diverse boiling times produce different polyphenol profiles in foods, and a long time can cause more severe damage than a short one [62]. The type of heat treatment affects the polyphenol bioavailability. Steaming is the best heat method to preserve phenolic fractions since they are indirectly exposed to water [63]. The form in which phenolics are present also affects bioavailability [64].

Canning, a process employed to produce sterilized and microbiologically safe food products by applying heat treatment, can decrease phenolic compound levels [65] because they migrate into the surrounding medium [66].

Drying, the preservation process that aims to decrease the moisture content of food by using heat and mass transfer, can affect the phenolic levels in the function of the temperature regime. Freeze-drying is the most efficient method to preserve phenolic content, while hot-air-drying is the least. The vast variety of chemical polyphenol classes also influences the variability in the effects caused by drying [67]. Oven-dried processes produce higher levels of bioaccessible phenolics than other drying processes [68,69,70]. Slow freezing enhances the bioavailability of the phenolic compounds since it forms ice crystals that favor the polyphenols extraction, oxidation, and degradation, during digestion [71].

Peeling fruits and vegetables determines the loss of high amounts of bioactive compounds since they are contained in the peel and external parts of the plants at higher levels than other parts [72]. Grinding, the technique that reduces the size of solid particles using mechanical forces, enhances the polyphenols extractions as a function of the particle size [73]. The ultrasound treatments pulsed electric field, high-pressure, and pulsed-light processing enhance polyphenols digestion, bioaccessibility, and bioavailability [74].

## 6. Polyphenol Biological Activities

Epidemiological studies have shown an inverse association between a polyphenolic-rich diet and the risk of chronic human diseases. Polyphenolic compound-rich foods and beverages can have antioxidant, anti-inflammatory, anticancer, and anti-aging properties and reduce the risk of degenerative diseases such as cardiovascular, diabetes mellitus, and neuronal diseases.

### 6.1. Antioxidant Activity

**Figure 2:**
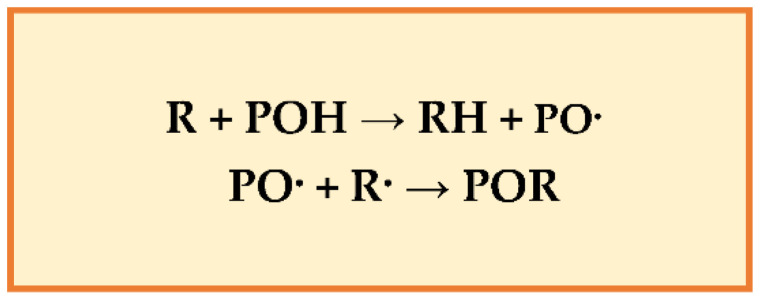
Reactions between lipids and phenols.

Experimental evidence showed that polyphenols protect cell constituents against oxidative damage and degenerative diseases associated with oxidative stress [75]. The polyphenol-rich foods can improve plasma antioxidant capacity by scavenging radical species (e.g., ROS, RNS) or repressing radicals’ formation by inhibiting the activities of the oxido-reductive enzymes’ and/or chelating the metals that intercept free radical production. Their phenolic groups can accept an electron to form phenoxy radicals, interrupting chain oxidation reactions, and conjugated aromatic systems can delocalize an unpaired electron [76]. Polyphenols reduce the oxidation of lipids and other molecules by donating hydrogen to radicals (R). The resonance makes PO· (phenoxy radical) relatively stable (new chain reactions are not started) and acts as terminators of the propagation route when reacting with other free radicals (Figure 2).

The reduction activity of phenolic acids and their derivatives depends on the free hydroxyl groups in the molecule [77]. The hydroxycinnamic acids show better antioxidant activity than hydroxybenzoic acid equivalents due to the aryloxy-radical stabilizing effect of the –CH=CH–COOH linked to the phenyl ring by resonance [78]. The phenolic acids’ antioxidant activity of free, esterified, glycosylated, and nonglycosylated phenolics is mainly ascribed to radical scavenging via the hydrogen atom donation mechanism [78,79].

The flavonoids’ radical scavenging depends on the ortho-dihydroxy structure in the B ring, which allows higher stability to the radical form and participates in electron delocalization of the 2,3-double bond with a 4-oxo function in the C ring [77].

The chemical electron deficiency of anthocyanins is particularly reactive toward ROS/RNS.

The polyphenolic compounds with dihydroxy groups prevent metals-induced free radical formation by conjugating the transition metals that interact with hydrogen peroxide (H_2_O_2_) through the Fenton reaction to form hydroxyl radicals (·OH).

Phenolic compounds with catecholate and gallate groups can stop metal-induced oxygen radicals by improving metal ion autoxidation or forming an inactive complex with weaker interaction [79].

The metal ions can attack the flavonoids into positions 3′ and 4′ (B ring), 3 and 4, 3 and 5, 4-keto and 3-hydroxy, and 4-keto and 5-hydroxy (C ring) [80].

Moreover, polyphenols can improve cellular antioxidant activity by regulating Nrf2, which controls some detoxifying enzymes (SOD, GSH, GPx1, NADP(H) quinone oxidoreductase, HO-1, and GST) [77]. Finally, polyphenols can influence microRNAs [81].

MicroRNAs are a class of small, endogenous, noncoding RNAs. Some microRNAs (i.e., miR-21, miR-125, and miR-146) are involved in vascular inflammation and diseases [82,83,84]. Dietary polyphenols can influence the microRNAs’ expression and biogenesis [30]. For example, curcuminoids can act as anti-atherosclerosis agents by upregulating miR-126 expression [85]. Resveratrol can act as a cardioprotective molecule by improving the mRNA activating SIRT1, and enhancing the SOD’ levels [86,87,88,89].

Gallic acid can decelerate atherosclerosis progression by upregulating miR-145 and downregulating miR-21 expression [90].

Under certain conditions, the polyphenolic compounds can initiate an autoxidation process and perform as prooxidants. In these cases, the phenoxy radicals can interact with oxygen to make quinones and superoxide anions [91]. pH, high concentrations of transition metal ions, and oxygen molecules can induce the autooxidation of polyphenols [92]. Quercetin and gallic acid can have prooxidant activity; instead, the hydrolyzable tannins have little or no prooxidant activity [93].

### 6.2. Anti-Inflammatory Activity

Plant polyphenols can decrease the effect of the cytokine, affecting their receptors or reducing their secretion processes [94].

Phenolic compounds can suppress the binding of proinflammatory mediators, control eicosanoid synthesis, prevent stimulated resistant units, and impede the activity of COX-2 and NO synthase, acting on NF-κB [95]. Some phenolic acids, such as rosmarinic acid and isosalvianolic acid, can reduce the production of IL-6, TNF-α, and IL-1β at the gene and protein levels [30].

The catechols’ enzymatic activity depends on the structure of the B ring and needs nucleophilic additions [96]. The procyanidins decrease the concentrations of NO, prostaglandin E2, and ROS [97].

The flavonoids (e.g., flavones) regulate IL-6 in the blood [98]. The flavonoids’ anti-inflammatory mechanism is related to the unsaturation in the C ring that affects the strength of binding interactions by resonance [99].

### 6.3. Anticancer Activity

Cancer development consists of initiation, promotion, progression, invasion, and metastasis [100]. Genetic mutations occur when DNA damage is not repaired, and a clone of mutated cells is reproduced during mitosis. Tumor promotion is a reversible and long-term process in which a selective clonal expansion of the cells forms a population of aggressively proliferating multi-cellular cells (premalignant tumor). Clonal expansion determines the development of the premalignant cells into tumors (tumor progression phase). Finally, some tumor cells may be cut off from the primary tumor mass, migrate toward blood vessels or lymphatic vessels and produce a second lesion (invasion and metastasis phases). The natural phenolic compounds can induce cell cycle arrest at G1, S, S-G2, and G2 phases by down-regulating cyclins and cyclins-dependent kinases or producing the expression of p21, p53, and p27 genes [80].

Polyphenols can act against tumor initiation and promotion, changing the redox status and affecting essential cellular functions (i.e., cell cycle, apoptosis, angiogenesis, inflammation, invasion, and metastasis) [101]. Oxidative damage can cause cancer since ROSs can damage the DNA and affect cell replication and signal transduction [102].

The flavonoid anticancer effects are related to their antioxidant and pro-oxidant activities [103,104]. The flavonol kaempferol can induce apoptosis and arrest in the S-phase of cancerous cells by modulating ROS levels [105]. When it acts as pro-oxidants it decreases NF-κB levels and produces cyclooxygenase-2 (COX) overexpression, inducing apoptosis, and cell-cycle arrest [106].

Some flavonoids and resveratrol can affect the procarcinogens’ activation by impeding phase I metabolizing enzymes (e.g., cytochrome P450) [107,108,109,110]. They can help carcinogens’ detoxification and removal inducing the phase II metabolizing enzymes (e.g., glutathione S-transferase, UDP-glucuronyl-transferase, and NAD(P)H quinine oxidoreductase) [111].

The polyphenols can produce apoptosis-inducing cell cycle arrest inhibiting the extracellular regulated kinase, c-Jun *N*-terminal kinase, and P38 mitogen-activated protein kinase pathway, transcription factors, NF-κB, activator protein-1 (AP1), protein kinase C (PKC), and growth factor-mediated pathways. The apigenin inhibits the growth of human thyroid carcinoma cells, probably by decreasing the phosphorylation of MAPK and by activating the protein kinases, and scavenging H_2_O_2_ [112].

The 3,4 dihydroxybenzoic acid stimulates apoptosis, in human gastric carcinoma cells, by ROS overproduction which can activate JNK/p38 MAPKs [113].

The polyphenolic compounds can negatively affect some factors involved in the inflammatory processes, such as the NF-κB, proinflammatory cytokines release, COX-2, lipoxygenases, inducible nitric oxide synthase, and MAPK-mediated pathway [80]. For example, Epigallocatechin gallate can block NFκ B activation in human epithelial cells and downregulate the expression of inducible nitric oxide synthase and nitric oxide production in macrophages [114]. Finally, the kaempferol can counteract malignant cell invasion and metastasis, down-regulating the matrix metalloproteases (MMP-2 and MMP-9), urokinase-plasminogen activator (uPA), and uPA receptor expression [115,116].

### 6.4. Cardiovascular Protective Activity

Cardiovascular (CVD) pathologies are the primary cause of morbidity and mortality (ischemic heart disease and stroke contribute 85%) [117]. Oxidative stress and inflammatory processes are considered promoters of endothelial dysfunction [118,119]. Polyphenols have antioxidant, anti-inflammatory abilities and can modulate lipid metabolism [120]. They (mainly quercetin and resveratrol) can decrease LDL oxidation [121], cholesterol synthesis, improve LDL receptor expression and activity [122,123], and the cholesterol transporters expression [124]. The anthocyanins and resveratrol can improve fecal cholesterol elimination [125,126] and decrease the triglyceride plasma level, decreasing the apolipoprotein B48 and apolipoprotein B100 production in the liver and intestine [127] or the lipoprotein lipase expression [128]. Flavonoids can also reduce blood pressure ameliorating flow-mediated dilation in humans (by improving the NO synthase activity) [129,130] and influencing the renin-angiotensin system [131,132]. Moreover, they can prevent platelet aggregation, decreasing the activity of cyclooxygenase 1, and thromboxane A2 that act as a vasoconstrictor and platelet aggregation’s inducer, respectively [133]. Finally, the polyphenols’ prebiotic-like activity can account for the amelioration of markers of CVD [124].

### 6.5. Antidiabetic Activity

**Figure 3:**
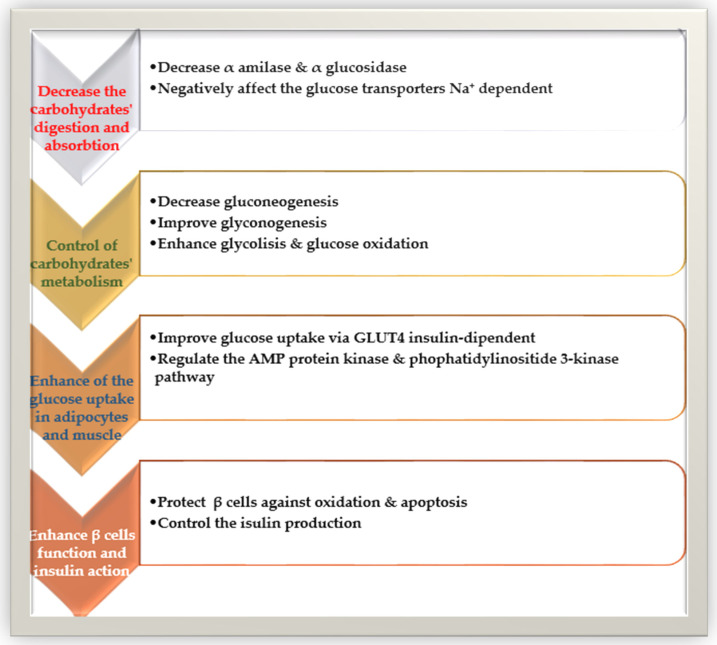
The polyphenols’ effects on glucose homeostasis and insulin resistance.

There are two main types of diabetes (diabetes-1 and diabetes-2). Diabetes type-2 or diabetes mellitus is due to damage in glucose metabolism and advancing insulin resistance that leads to hyperglycemia. The leading causes of hyperglycemia are dietary carbohydrates’ digestion and absorption, glycogen storage reduction, β-cell dysfunction, peripheral tissue insulin resistance, deficiency in insulin signaling pathways, and improved gluconeogenesis and production of hepatic glucose [134]. Polyphenols can decrease the intestinal absorption of carbohydrates, control the enzymes that regulate glucose metabolism, and increase the β-cell functionality, insulin secretion, and the anti-inflammatory and antioxidant properties of these components (Figure 3).

The phenolic acids, flavonoids, and tannins can regulate the key enzymes responsible for the digestion of carbohydrates (α-glucosidase and α-amylase) [135]. The catechin, epicatechins, and chlorogenic, caffeic, ferulic, and tannic acids can decrease the glucose transporters Na^+^-dependent (SGLT1 and SGLT2) [136]. The coffee phenols, anthocyanin, and curcumin can regulate postprandial glycemia and decrease the progression of glucose intolerance by a simplified insulin response and improved secretion of glucagon-like polypeptide-1 (GLP-1) and glucose-dependent insulinotropic polypeptide (GIP) [137]. Ferulic acid can decrease blood glucose by improving glucokinase activity and glycogen production in the liver [138]. Catechins and epicatechins can decrease hyperglycemia and hepatic glucose output, downregulating the expression of liver glucokinase, and upregulating the glucose-6-phosphatase and phosphoenolpyruvate carboxykinase [139].

### 6.6. Neurodegenerative Protection

Neurodegenerative diseases are due to the deterioration of neurons’ structure and/or function. Reactive oxygen and reactive nitrogen species can determine neuronal cell dysfunction and death. The phenolic compounds can interact with the amino acid residues of acetylcholinesterase’s (AChE) active site, making hydrogen bonds and hydrophobic and π–π interactions. Multiple hydroxyl groups can improve the inhibition of AChE, increasing the binding capacity [30]. Resveratrol can protect against microglia-dependent β-amyloid toxicity by decreasing the nuclear factor κB [140]. Some polyphenols protect against Parkinson’s disease by scavenging the neurotoxin N-methyl-4-phenyl-1,2,3,6-tetrahydropyridine (MPTP)-mediated radical formation [141] or decreasing free radicals’ formation by chelating iron [142].

### 6.7. Anti-Aging Action

Aging determines detrimental changes in the cells and tissues. The cosmetic industry constantly strives in product development and reformulation to meet consumers’ preferences. Today, nature-derived products are in demand on the market. Some botanical preparations that contain polyphenols (e.g., flavonoids, phenolic acids, and stilbenes) are employed in the composition of anti-aging products [143]. Free radicals and oxidative stress are the major contributors to aging damage. The phenolic hydroxyl groups on polyphenol molecules can scavenge ROS [144]. The polyphenolic compounds can regulate the production of oxidase enzymes (sodium oxide dismutase 1 in the cytosol, sodium oxide dismutase 2 in the mitochondria), and endogenous antioxidants [145,146,147,148,149] can improve the transcriptional factor Nrf2 DNA-binding activity and regulate protein expression [150,151,152]. Anti-aging formulations contain botanicals metabolites able to protect DNA, regulate the enzymes’ action, decrease inflammation, and alter hormone imbalance [143].

The epigallocatechin-3-gallate in green tea decreases the UVB-induced hydrogen peroxide release from normal epidermal keratinocytes, MAPK phosphorylation, and inflammation by activating NFkB. The flavins in black tea decrease UVB-induced AP-1 induction, prevent UVB-induced phosphatidyl-inositol 3-kinase activation, decrease the amount of ROS in the skin, and offer photoprotection by reducing local and systemic immunosuppression UVB-induced [153]. Resveratrol is employed to reduce hydrogen peroxide, improve lipid peroxidation, and decrease the levels of COX-2 and ornithine decarboxylase. Moreover, it can decrease UVA-induced oxidative stress in human keratinocytes since it controls the Keap1-a protein that acts on Nrf2 [153]. Curcuminoids (found in Turmeric spice) can decrease inflammation by inhibiting the MAPK and NFkB signaling pathways and decreasing nitric oxide levels and COX2. Moreover, in keratinocytes and fibroblasts, curcuminoids can decrease UVB-induced TNF mRNA expression and matrix metalloproteinase-1 expression [154].

### 6.8. Antiallergic Action

Allergic diseases happen when an organism becomes sensitive to an innocuous allergen and releases many allergy-related intermediaries. Polyphenols limit the production of IgE, the release of allergic mediators, and allergy symptoms. Polyphenols can control hypersensitivity by regulating oxidation and interacting with inflammatory mediators [155]. Catechins can decrease Th2 cytokine production and T cell activation and proliferation. Caffeic, chlorogenic, and ferulic acids can irreversibly bind peanut allergens (Ara h1 and Ara h2), reducing their allergenicity [156]. Punicalagin, phloridzin, and rutin can improve the growth of probiotics such as *Lactobacillus* and *Bifidobacterium*, which positively impact food allergies [157].

### 6.9. Antiosteoporotic Action

Osteoporosis causes the loss of bone mineral density, decreased bone mass, and microstructural deterioration. Flavonoids and stilbenes can improve osteogenesis by controlling the bone morphogenetic protein, NF-κB, IGF, and MAPK, and can inhibit the osteoclastogenesis pathways through epigenetic regulations. They can activate SIRT-1 (histone deacetylase) and modify the NAD^+^/NADH ratio [158,159,160].

### 6.10. Antimicrobial Action

Some plant extracts rich in polyphenols can decrease the growth of fungi and bacteria (i.e., Listeria monocytogenes, *Salmonella* spp., and *Escherichia coli*) [161,162], minimize the exposure of humans to resistant bacteria [163], and can have a synergic action with other antimicrobials. These findings have suggested a potential use of polyphenol-rich extracts as food preservatives and in the pharmaceutical industry to improve efficacy and decrease antibiotic side effects, such as repressing antibiotic-resistant bacteria [164]. The polyphenol-rich extracts can be placed on the food surface by spraying, dipping, brushing, or mixing with other ingredients [165]. Unfortunately, in some cases, the interaction with food components can cause a lack of antimicrobial efficacy. Therefore, it was thought to encapsulate them in carriers to increase their distribution in the food and reduce contact with food matrix molecules that reduce their effectiveness [166]. The mechanisms of antibacterial action are not yet entirely deciphered. However, it is known that many sites of action at the cellular level are involved. Polyphenols can modify the cell membrane permeability, destroy the cell wall integrity and change intracellular functions by binding some enzymes [167].

## 7. Polyphenols Potentialities in the Nutraceutical Era

Today, consumers include a high level of bioactive compounds in their standard diet, preferably derived from natural sources such as plants and fruits, in the hope of giving more life to the years by preventing disabling pathologies that decrease the skills that allow living life in all its manifestations [168]. Food and pharmaceutical companies develop nutraceutical foods and supplements that contain botanical extracts and metabolites alone or combined with other ingredients [169,170,171].

Numerous studies strongly suggested that including polyphenols or polyphenol-rich extracts in supplements or foods may protect the body tissues against oxidative stress and aging [172,173].

The primary issue in using plant extracts is that fungi, which can produce toxins, can contaminate the extracts (e.g., *Aspergillus* section Nigri that produces Ochratoxin A, a carcinogenic, teratogenic, nephrotoxic, neurotoxic, and immunotoxic toxin) [174]. The research should implement efforts to develop analytical controls that safeguard consumer safety.

## 8. Polyphenols Extraction

**Figure 4:**
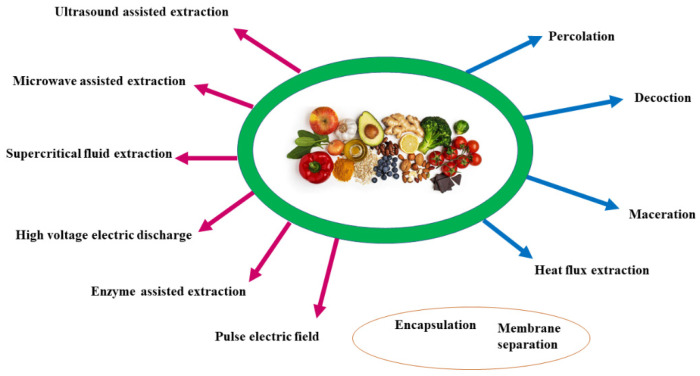
The polyphenols’ traditional and modern extraction methods. Blue arrows: traditional extraction methods; red arrows: modern methods; in the orange circle: techniques used to improve polyphenol management.

Extraction plays a pivotal role in the purification of polyphenols from foodstuffs. Extraction techniques can employ traditional or “conventional” such as percolation, maceration, and Soxhlet extraction, and modern methods, such as ultrasound or microwave, the latter is most extensively used (Figure 4). In both cases, extraction efficiency depends on various factors such as the nature of the solvent, solvent–solid ratio, temperature, and particle size. Polyphenol can be extracted from fresh, frozen, or dried plant samples. The extracts can be added to an organic solvent, such as methanol or ethanol, with low viscosity to accelerate mass transfer [175]. Before extraction, the pretreatment of the plant matrices (e.g., cleaning, washing, milling, grinding, drying, homogenizing) is crucial.

Percolation extraction uses water as a solvent. It takes a long time to obtain the pure extract to be concentrated [176].

The maceration is a solid-liquid extraction method using different solvents depending on the target compounds’ physical and chemical properties. It has low efficiency and extraction yield and employs a large volume of solvents compared to non-conventional techniques such as ultrasound-assisted extraction (UAE) [177]. A higher ratio of solid/solvent increases polyphenols recovery [178].

Decoction extracts plant materials by boiling. It is inefficient for heat and light-sensitive compounds [179]. The decoction of Citrus fruits produces by-products of Citrus peels with high concentration levels of polyphenol fraction [180].

Heat reflux extraction is a solid–liquid extraction method performed with repeated solvent evaporation and condensation at a constant temperature. It requires less extraction time and solvent than percolation or maceration and allows for a greater extraction yield [181]. Polyphenols from wastes of *Vitis vinifera* were extracted by Moldovan et al. by using heat reflux extraction [182].

The solid-phase extraction (SPE) method is considered quick and easy to extract polyphenols from vegetable oils. According to the experimental needs, different stationary phases were used (e.g., C8 cartridges, octadecyl C18, diol-bonded phase cartridges, amino-phase cartridges, and octadecyl C18EC) [6]. The non-conventional techniques employ supercritical fluid, high-voltage electric discharge, and enzyme-assisted extraction. It is preferable to extract the bound polyphenols, also referred to as non-extractable polyphenols (NEP), from plant sources using one or more combinations of modern technology rather than conventional methods. A comparison between traditional and SFE extraction performed on black tea leftovers showed that SPE technique gives the best performance in extraction of phenolic compounds (SPE gives 521 mg GAE/g; traditional gives 283 mg GAE/g) [183].

The extraction with supercritical fluids such as CO_2_, propane, argon, and SF6 allows for an easy penetration inside plant materials and high solvents. Power-pulsed electric fields (PEF) perform a gentle extraction due to the electroporation of cell membranes. It has been applied to exotic fruits, grapes, and oil crop components [184]. Microwave-assisted extraction (MAE) is an eco-friendly technique with higher efficiency in the recovery of polyphenols from waste products if compared to that of extracts prepared by ultrasound-assisted extraction (UAE) and conventional methods such as maceration [185].

Fruit skins, stem barks, grain seed coats, brans, and pods, are generally considered agro-waste, but they are essential resources for NEP recovery [186,187,188]. Table 1 reports conventional and non-conventional techniques applied to extract polyphenols from organic waste.

## 9. Polyphenol Nano Delivery Systems

The potential of antioxidative and repair pathways decreases with age, causing several adverse effects such as the risk of neurodegenerative diseases such as Parkinson’s disease, memory loss, Alzheimer’s disease, atherosclerosis, and cancers due to the accumulation of reactive oxygen species. Polyphenols, due to their oxidizing ability, can protect from the damaging effects of ROS. Therefore, they can be used as active compounds in several formulations preventing oxidative stress [197,198]. The loading of polyphenols into lipid nanocarriers (NCs) is an essential tool for increasing bioavailability, reducing degradation, and protecting antioxidant polyphenols’ activity. The NCs are biodegradable and have no significant toxicity. The nanoemulsion, liposome, phytosome, solid lipid nanoparticles (SLNs), nanostructured lipid carrier (NLCs), and lipid-polymer hybrid nanoparticles (LPHNs) can encapsulate polyphenols to improve their biophysiological target [199]. Nanoparticles (NPs) have diameters as small as 1–100 nm. They can enhance polyphenols delivery and promote their absorption and bioavailability [200]. The transcellular pathway is a route for the NPs transportation (via endocytosis or macropinocytosis mechanisms) and subjected to the degradative microenvironment of the cellular lysosome environment in the acidic endosomal lumen [201]. Carbohydrate-based delivery systems, such as mono, oligo, and polysaccharide, are employed to encapsulate polyphenols due to their abundance and low cost; for example, curcumin was encapsulated in chitosan using an injection-gelation method to increase its bioavailability, antioxidant properties, and to improve stability and effects on tumor cells [202].

Protein-based systems have been employed to prepare nanoparticles for carrying polyphenols. The proteins act as “host” and polyphenols as “guest” molecules. The polyphenols bind specific regions on the protein surfaces through hydrogen and/or hydrophobic non-covalent bounds. The β-lactoglobulin nano delivery was used to increase the water solubility 3-fold at pH 7 of the epigallocathechin-3-gallate and naringenin [203]. Finally, polyphenols are used to design polyphenol-based nanomaterials for biomedical applications. For example, the polyphenol-grafted polymers are used as antidiabetic agents [204], the curcumin encapsulated in chitosan and polyglycolic acid (PGA) particles for wound healing [205], and the polyphenol-loaded electrospun nanofibers to improve the remineralization and regeneration of bone [206].

## 10. Polyphenols in Active Packaging

In recent years, packaging technology has evolved, including intelligent or smart packaging. Biodegradable, active, and bioactive packaging are new trends in food packaging research. Food contact materials (FCM) are engineered to protect foods, and improve their shelf-life [207]. Plastic packaging materials, such as polycarbonate, polyethylene, and polyethylene terephthalate, widely used in food packaging, are nonbiodegradable and disadvantageous to the environment and human health [208,209,210]. To date, natural and biodegradable biopolymer-based packaging films and edible coatings represent the alternative to plastic packaging materials [211]. Natural biopolymers, including proteins, polysaccharides, and lipids, have been used in packaging manufacturing [212]. Fruit industrial manufacture generates large amounts of waste that harm the environment and causes considerable treatment expense [213]. Nevertheless, these by-products are rich in bioactive compounds [214], some of which can be incorporated into biodegradable plastics for food packaging to protect the polymeric matrix against thermal [162,213], photo-induced degradation, and preserve the food freshness and quality [215]. The solvent-based impregnation of biodegradable polymers with extracts of *Cistus linnaeus* is used to improve the polymers’ thermal stability [216]. Chemically-synthesized and biomass-derived biodegradable polymers have been used as matrices to protect food during transportation, storage, and sale. Polyphenols are employed in the so-called “leaching systems” that are active-releasing systems able to release actives by direct contact between food and packaging material. For this purpose, the propolis is mixed with biopolymers, plasticizers, and reinforcing agents to produce active packaging and edible coatings [213,216].

Biopolymers can be dissolved in solvents depending on their hydrophilicity. Hydrophilic biopolymers, such as gelatin, κ-carrageenan, alginate, and agar in water, chitosan, can be dissolved in acidic solutions [217], while organic solvents, chloroform, and ethyl acetate can be employed for hydrophobic biopolymer such as polylactic acid (PLA) [218]. For example, the ethanolic propolis extract, and propolis in powder, together with plasticizers such as glycerol and (PEG) polyethylene glycol, have been added to biopolymeric solutions [219]. Tea polyphenols (TP) are employed as an active component in biopolymer materials for active food packaging. Shao et al. have incorporated TP into pullulan-carboxymethylcellulose sodium (Pul-CMC) solutions on electrospun nanofiber films [220].

## 11. Conclusions

Polyphenols are secondary plant metabolites that can benefit human health and preserve food. The use of polyphenols as supplements, antibiotic drugs, cosmetics, and natural food preservatives is a promising trend in the industry because of the growing interest in natural products and the multiple biological activities of these products.

The great demand for polyphenols and the small quantity produced by the plants have determined the need to use extraction techniques that allow exhaustive extraction even when it is necessary to recover them from non-traditional sources such as organic waste. For this purpose, unconventional techniques such as supercritical fluid, high-voltage electric discharge, and enzyme-assisted extraction must be optimized.

The polyphenols’ low oral bioavailability and interactions with other molecules negatively impact the possible industrial application. Therefore, different nanocarriers have been developed to protect, improve bioavailability, ensure achievement to the active site, and improve their effectiveness. It is essential to underline that polyphenols are commonly present in plant-based foods such as fruits and vegetables.

Guidelines for their consumption and supplementation should be provided by regulatory bodies to make consumers safe and informed.

## Figures and Tables

**Table 1:** Extraction of free and bound polyphenols.

Conventional Extraction Methods	Matrix	Extraction Solvent/Membrane Separation Type	Higher % of Recovery Compared to Traditional Method	Reference
Percolation	*Vernonia cinerea leaves*	Ethanol 60%		[188]
Decoction	*Citrus peels*	Ethanol 75%		[180]
Heat reflux extraction	*Pleioblastus amarus*	Ethanol 75%		[189]
Maceration	*Citrus peel*	Ethanol 80%		[180]
**Non- Conventional methods**				
Ultrasound assisted	*Olive pomace*	water	58%	[190]
Microwave assisted	*Blackcurrant By-Products*	water	25%	[191]
Supercritical fluid	*Lees Vitis vinifera grapes*	CO_2_	−35%	[192]
High voltage electric discharge	*Spent coffee grounds*	24% ethanol	20.03%	[193]
Pulse electric field	*Vitis vinifera, Sideritis scardica and Crocus sativus*	water	44.36–49.15%	[194]
Enzyme assisted	*Green yerba mate*	water	38.67–52.08%	[195]
Membrane assisted pre-purification	*Winery and olive mill wastes*	NF270 membrane	95% for polyphenol compounds removal	[196]

## Data Availability

Not applicable.
